# Editorial

**Published:** 1870-12

**Authors:** 


### Editorial.

***Publishers' Notice.***

Readers of the JOURNAL will not fail to recollect that this is tne last No. of the volume, and that continuance of their subscriptions necessitates payment in advance. Prompt remittance will enable us to determine the size of the edition to be published, and, if as general as we have every reason to expect, will permit liberal improvements in every department included. A glance at the general index of the volume, now complete, will show tl.e range and variety of topics noticed and discussed, and that few or none of the matters about which the profession have been thinking and writing are neglected. In the ensuing volume, space will be gained for greater attention to many practical subjects by condensation of our foreign department. The elaborate papers on physi ological subjects, which have constituted an important feature of the present volume, whilst they have elicited the warmest interest of a goodly number of our readers, yet have detracted somewhat from the immediately practical character which we are desirous hereafter of impressing upon the JOURNAL. We must remit these elaborate investigations and discussions to the monographs, the quarterlies, and cyclopædias. The gist of the matters observed, discussed and discovered, will be given, and all reasonable latitude permitted to correspondents in their efforts to arrive at and develop medical truth. But the Editors have determined upon what they conclude best for the interests of both the JOURNAL and subscribers, to make it more than ever a thorough abstract and chronicle of the time—a necessity for each and every practicing physician who desires to keep pace with the steady, onward march of the profession.

On the part of the Publishers no care or expense will be spared to make its mechanical appearance creditable to themselves, and its prompt issue satisfactory to subscribers.

***The Editors***

Pledge to their readers, for the coming year, increased contributions of time and industry to the highest development of the important organ of the profession committed to their charge. An enlarged corps of contributors has been secured, and every effort will be put forth to make the JOURNAL a welcome visitant to the tables of physicians, and a valuable assistant to them in solving the professional problems of their chosen work.

***Palato-Pharyngeal Tumor.***

In the November No. of the JOURNAL (p. 653), Dr. Chesbrough reports from the Surgical Clinic of Prof. Gunn, the removal of an extraordinary tumor from its attachment to the *velum pendulum palati.* So far as the standard books are concerned, the case is unique, and certainly the difficulties attending its removal can scarcely be exaggerated in description. The fortunate result may well be considered a surgical triumph.

In the *Southern Medical and Surgical Journal*, May, 1853, we find the report, by Dr. L. A. Dugas, of a case of fibrous tumor in the same locality, which he successfully extirpated.

Of this tumor, Dr. Dugas says: "The soft palate was carried forwards and downwards, so as to constitute a prominence the size of a large egg, to the posterior surface of which [the palate] the tumor was attached. Deglutition was so difficult that he could take no solid food, his articulation was very indistinct, and respiration considerably impeded when he would walk briskly, causing him to breathe loudly, and like a horse affected with the 'bellows.'"

The operation instituted by Dr. Dugas, however, was much more formidable than that described by Dr. Chesbrough as succeeding in Dr. Gunn's case. He says:

"Provided with actual cauteries, a syringe, sulphate of zinc, etc., to control the hemorrhage from the general surface and smaller vessels, I passed a ligature beneath the right carotid artery, and left it there, ready to be tied, should this become necessary. The patient was then seated in a chair, and an incision made from the right angle of the mouth to the masseter muscle, which necessitated the ligature of the facial artery. In the third stage of the operation, a longitudinal incision was made from the side of the uvula to the roof of the mouth, through the soft palate."

In this manner the tumor was removed from its attachments, the strong connective tissue being torn by the fingers. In Dr. Dugas' case there was also found a small supplementary tumor, apparently in connection with, and pressing down, the right tonsil, which had not been discovered until after the removal of the larger tumor. This was also removed in a similar manner, although not without considerable difficulty. The patient fully recovered. Subsequent microscopic examination showed the tumors to be purely fibrous. We regard it as better to adopt the plan of Prof. Gunn, and have this examination made prior to the operation.

***Free Advertisement.***

CHICAGO, September 30, 1870.

J. L. SMITH, Esq.—DEAR SIR: From a careful examination of the "Home Bitters," and the formula from which they are manufactured, we have no hesitation in saying they are a most excellent tonic, and we are satisfied they are equal, if not superior, to any bitters yet offered to the public. Yours respectfully,

R. LUDLAM, M.D., 297 Wabash Ave.,
Prof. of Obstetrics and Diseases of Women and Children, and Dean of Faculty, Hahnemann Medical College.

TEMPLE S. HOYNE, A.M., M.D., 711 Wabash Ave.,
Prof. of Materia Medica and Therapeutics, Hahnemann Medical College.

And our friend Mariner, "Analytical Chemist," etc., certifies that the "bitters" are in general use by the medical profession—in this preparation presented in pure alcoholic solution. As the reporters say, "comment is unnecessary."

***Freedom of Opinion.***

It is perhaps unnecessary for us again to announce that we do not hold ourselves responsible for the opinions of our correspondents. Truth is not a mere matter of opinion, and there is a world of sense in the old observation that men rarely come at a reasonable opinion on any subject until they have first exhausted all the absurd views it is possible to take of it. All we ask is, that our correspondents shall have intelligent notions, and advance them in good faith.

---

DOCTOR LYMAN STANTON, of Copenhagen, N. Y., tells the following good story—true, and to the point: "A gentleman who had just lost a member of his family under the treatment of poisons, said to his physician, 'This is the last doctor's bill that I shall ever pay; I shall never employ a doctor again so long as I live and have my senses.' The doctor replied, 'Drowning men catch at straws, and you may yet be glad to see the doctor.' The gentleman replied, '*No, never!* I shall trust in *God Almighty* and *catnip tea.*'"—*Eclectic Review.*

